# Regulation of DMD pathology by an ankyrin-encoded miRNA

**DOI:** 10.1186/2044-5040-1-27

**Published:** 2011-08-08

**Authors:** Matthew S Alexander, Juan Carlos Casar, Norio Motohashi, Jennifer A Myers, Iris Eisenberg, Robert T Gonzalez, Elicia A Estrella, Peter B Kang, Genri Kawahara, Louis M Kunkel

**Affiliations:** 1 Program in Genomics and Division of Genetics, Children's Hospital Boston, 3 Blackfan Circle, CLS15024, Boston, MA 02115, USA; 2 Department of Pediatrics and Genetics, Harvard Medical School, 25 Shattuck Street, Boston, MA 02115, USA; 3 The Manton Center for Orphan Disease Research, Children's Hospital Boston, 300 Longwood Avenue, CLS15031 Boston, MA, USA; 4 Harvard Stem Cell Institute, Holyoke Center, Suite 727 W, 1350 Massachusetts Avenue, Cambridge, MA 02138, USA; 5 Department of Neurology, Children's Hospital Boston and Harvard Medical School, 300 Longwood Avenue, Boston, MA 02115, USA

## Abstract

**Background:**

Duchenne muscular dystrophy (DMD) is an X-linked myopathy resulting from the production of a nonfunctional dystrophin protein. MicroRNA (miRNA) are small 21- to 24-nucleotide RNA that can regulate both individual genes and entire cell signaling pathways. Previously, we identified several mRNA, both muscle-enriched and inflammation-induced, that are dysregulated in the skeletal muscles of DMD patients. One particularly muscle-enriched miRNA, miR-486, is significantly downregulated in dystrophin-deficient mouse and human skeletal muscles. miR-486 is embedded within the *ANKYRIN1(ANK1) *gene locus, which is transcribed as either a long (erythroid-enriched) or a short (heart muscle- and skeletal muscle-enriched) isoform, depending on the cell and tissue types.

**Results:**

Inhibition of miR-486 in normal muscle myoblasts results in inhibited migration and failure to repair a wound in primary myoblast cell cultures. Conversely, overexpression of miR-486 in primary myoblast cell cultures results in increased proliferation with no changes in cellular apoptosis. Using bioinformatics and miRNA reporter assays, we have identified platelet-derived growth factor receptor β, along with several other downstream targets of the phosphatase and tensin homolog deleted on chromosome 10/AKT (PTEN/AKT) pathway, as being modulated by miR-486. The generation of muscle-specific transgenic mice that overexpress miR-486 revealed that miR-486 alters the cell cycle kinetics of regenerated myofibers *in vivo*, as these mice had impaired muscle regeneration.

**Conclusions:**

These studies demonstrate a link for miR-486 as a regulator of the PTEN/AKT pathway in dystrophin-deficient muscle and an important factor in the regulation of DMD muscle pathology.

## Background

Duchenne muscular dystrophy (DMD) is a progressive, X-linked, muscle-wasting disease that occurs in 1 in 3,500 live male births [1]. Males with inactivating mutations in the *DMD *(dystrophin) gene make a nonfunctional protein which results in degenerating skeletal muscles, severely elevated creatine kinase (CK) levels, cardiac arrhythmias and secondary infections and subsequent respiratory failure due to the degeneration of the diaphragm muscles [2,3]. By contrast, patients with Becker muscular dystrophy (BMD) also have mutations in the *DMD *gene, but produce a partially functional dystrophin protein [3]. Patients with BMD have mildly progressive muscle weakness and elevated CK serum levels as well as variable longevity and mobility [3]. While the genetic mutations that cause DMD are now well established, the subsequent steps that lead to the disease pathogenesis are still emerging [4]. In dystrophin-deficient muscles, it has been demonstrated that the platelet-derived growth factor (PDGF) receptor (PDGFR) signaling pathway is significantly altered [5,6]. Activation of PDGF signaling has been shown to promote the proliferation of myoblasts in primary myoblasts and C_2_C_12 _cultures [7]. PDGF signaling in skeletal muscles goes through a phosphatase and tensin homolog deleted on chromosome 10/AKT (PTEN)/AKT signaling pathway that is an important regulator of insulin signaling and myoblast differentiation, proliferation and cellular viability [8]. Recent studies in the *mdx *(dystrophin) mutant mouse model have found that *mdx *muscle contains elevated PTEN/AKT mRNA levels [9,10]. In addition, spontaneously occurring animal models of dystrophin deficiencies, such as the Golden Retriever muscular dystrophy dog, have been found to have elevated levels of PTEN within affected skeletal muscle fibers [11]. Thus, in two animal models of dystrophin deficiency, along with isolated biopsies from human DMD patients, the upregulation of PTEN/AKT signaling has been shown to be a secondary consequence of the lack of a functional dystrophin protein resulting from an as yet unclear mechanism.

Recently, we have shown that distinctive patterns of microRNA (miRNA) expression are associated with different types of primary muscle disorders [12]. One particular miRNA, miR-486, was significantly reduced in patients with DMD relative to control muscle biopsies. Intriguingly, this miRNA was reduced relative to control samples only in patients with DMD and not in those with the milder but allelic BMD. miRNA are known to be powerful regulators of multiple cell signaling pathways at critical time points in mammalian development and differentiation. miRNA can downregulate gene expression through either mRNA target degradation or inhibition of mRNA translation at the ribosome [13]. In skeletal muscle development, a series of muscle-enriched miRNA control myoblast differentiation and overall muscle strength and contraction [14].

Ankyrin 1 belongs to a family of proteins that contain the ankyrin repeat domain, which is essential for cellular membrane integrity and modulation of extracellular signaling factors [15]. mIR-486 is transcribed within the *ANK1 *(previously referred to as ankyrin-R) locus, from which both erythroid and skeletal muscle-specific isoforms are transcribed [16]. The smaller isoform of the *ANK1 *gene, *ANK1.5 *(also referred to as *sANK1*) contains an alternately spliced, muscle-specific exon (exon 39A) and lacks the characteristic ankyrin repeat domain [16,17]. ANK1.5 is a small, skeletal muscle-specific protein that associates with the sarcoplasmic reticulum of skeletal muscle myofibers [16,18]. Molecular interaction experiments revealed that ANK1.5 interacts directly with obscurin at the Z-disks of striated muscle fibers [19].

In a recent study, miR-486 was identified as a negative regulator of PTEN expression levels in muscle and subsequently in the PTEN/AKT pathway in normal cardiac and skeletal muscle development [20]. Using computational bioinformatics, we identified additional miR-486 downstream targets in adult skeletal muscle: PDGFRβ, PIK3R1 (p85α) and insulin-like growth factor 1 (IGF-1). Inhibition of miR-486 using a GFP-tagged lentivirus impaired cellular migration to a scratch wound injury, decreased myoblast fusion and increased cellular mitosis. Conversely, overexpression of miR-486 resulted in faster closure of the scratch wound, with no detrimental cellular apoptosis, compared to a scrambled miRNA control. Generation of muscle-specific miR-486-transgenic mice revealed perturbed muscle regeneration following cardiotoxin (CTX)-induced tibialis anterior (TA) muscle injury and dysregulation of the downstream target genes of miR-486 *in vivo*. Together these studies begin to characterize a functional role for miR-486 in normal and dystrophin-deficient skeletal muscle and further identify several components of the PTEN/AKT signaling pathway as potential targets for miR-486 regulation in muscle.

## Results and Discussion

### miRNA-486 expression is a significantly reduced miRNA in dystrophin-deficient skeletal muscle

**Figure 1:**
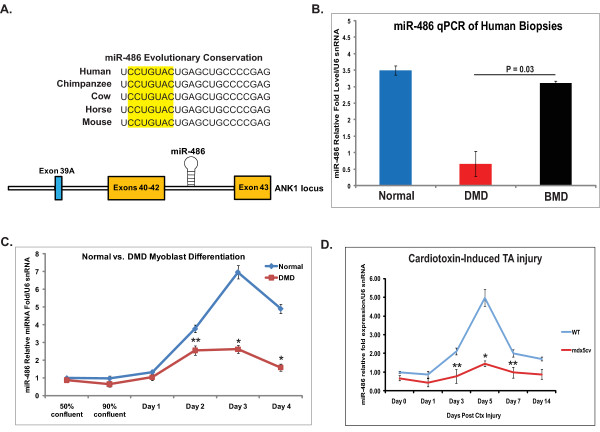
**miR-486 is a highly conserved mammalian miRNA that is differentially expressed in DMD skeletal muscle**. **(A) **miR-486 is conserved across mammals and is under the transcriptional regulation of the ankyrin 1 locus. The ANK1.5 isoform has a unique start exon (exon 39A; blue) that is transcribed independently of the ANK1.1 isoform and lacks the ankyrin repeat domain. **(B) **miR-486 is significantly reduced in DMD human skeletal muscle biopsies, but not in the milder BMD muscle biopsies, as compared to normal muscle controls. *P *= 0.03. The *y*-axis represents mature miR-486 fold levels normalized to U6 snRNA housekeeping controls. Error bars indicate SEM. Normal biopsies: *n *= 5, DMD biopsies: *n *= 5 and BMD biopsies: *n *= 3. **(C) **miR-486 is significantly upregulated during normal human myogenic differentiation, while the DMD myoblasts express miR-486 at significantly lower levels. The *y*-axis represents mature miR-486 fold levels normalized to U6 snRNA loading controls, and the *x*-axis represents the time of myogenic differentiation from 50% confluency (proliferating myoblasts) until day 4 differentiation (multinucleated myotube formation). At 90% confluency (day 0 differentiation), myoblasts were exposed to differentiation medium (2% horse serum). Blue lines represent normal human myoblasts, and red lines represent DMD myoblasts. **(D) **miR-486 is upregulated during skeletal muscle regeneration following CTX-induced TA muscle injury. Blue lines indicate wild-type C57B6/J mice, and red bars indicate *mdx^5cv ^*mice. The *y*-axis represents mature miR-486 fold levels normalized to U6 snRNA housekeeping controls. Error bars indicate SEM. *P < 0.005 and **P < 0.05.

Previously, we identified a biosignature of miRNA that was significantly dysregulated in human skeletal muscle biopsies taken from patients diagnosed with various muscular disorders [12]. One particular miRNA, miR-486, was significantly and specifically downregulated in DMD muscle, but not in patients with BMD. miR-486 is highly conserved among mammals and is embedded in the *ANK1 *gene locus within the intron between exons 41 and 42 (Figure 1A). Intriguingly, no miR-486 miRNA sequence has been identified within the genomes of nonmammalian species, such as fish or avians, although these species have the *Ank1 *gene sequence. To validate the miRNA microarray results indicating that miR-486 expression was significantly reduced in DMD muscles relative to control muscles, total miRNA was extracted from patients diagnosed with DMD or BMD or from normal controls (Figure 1B). Real-time quantitative PCR (qPCR) revealed that miR-486 expression was significantly reduced in the muscle biopsies from DMD patients, but not in those from BMD patients with a fully or partially functioning dystrophin protein (*n *= 5 control biopsies, *n *= 5 DMD biopsies and *n *= 3 BMD biopsies). In human myoblasts obtained from normal and DMD biopsies, miR-486 was expressed at similarly low levels early in myogenic differentiation (Figure 1C). However, as normal skeletal myoblasts progress along myogenic differentiation to form mature myotubes, miR-486 significantly increases until it reaches a plateau at day 3 of differentiation. In contrast, DMD myoblasts exhibit a slight increase in the expression levels of miR-486 during myogenic differentiation, but at significantly reduced levels compared to normal myoblasts, before miR-486's expression decreases by day 4 of myogenic differentiation (Figure 1C).

*In vivo *skeletal muscle regeneration can be induced following CTX injury and is widely used to study factors thought to be essential for new muscle fiber formation [21]. Wild-type and *mdx^5cv ^*(dystrophin-deficient) mice were injured in their TA muscles by injection of CTX. This resulted in the destruction of up to 90% of myofibers followed by the regeneration of new myofibers (*n *= 3 mice per time point for each cohort). Muscle biopsies from the injured wild-type and *mdx^5cv ^*mice were harvested, and total miRNA was extracted to determine whether the levels of miR-486 were dynamically regulated during skeletal muscle regeneration. CTX injury was validated by H & E staining of sections over the time course (representative images obtained on days 0 and 14 are shown in Additional file 1, Figure S1). In normal mouse muscle, miR-486 expression was significantly increased by day 5 after CTX injury, which is when nascent myotubes begin to appear [22,23] (Figure 1D). Conversely, *mdx^5cv ^*mice, which have impaired skeletal muscle regeneration following CTX injury [24], showed overall reduced levels of miR-486 during skeletal muscle regeneration, which were statistically significant (*P *< 0.005) at day 5 after CTX injury, when there is maximum miR-486 expression in normal mouse muscle (Figure 1D). Thus, miR-486 appears to be an important miRNA that is dynamically regulated during normal skeletal muscle regeneration, and its expression is significantly reduced in *mdx^5cv ^*mice.

### Inhibition of miR-486 in myoblasts and myotubes causes profound physical and cellular changes

**Figure 2:**
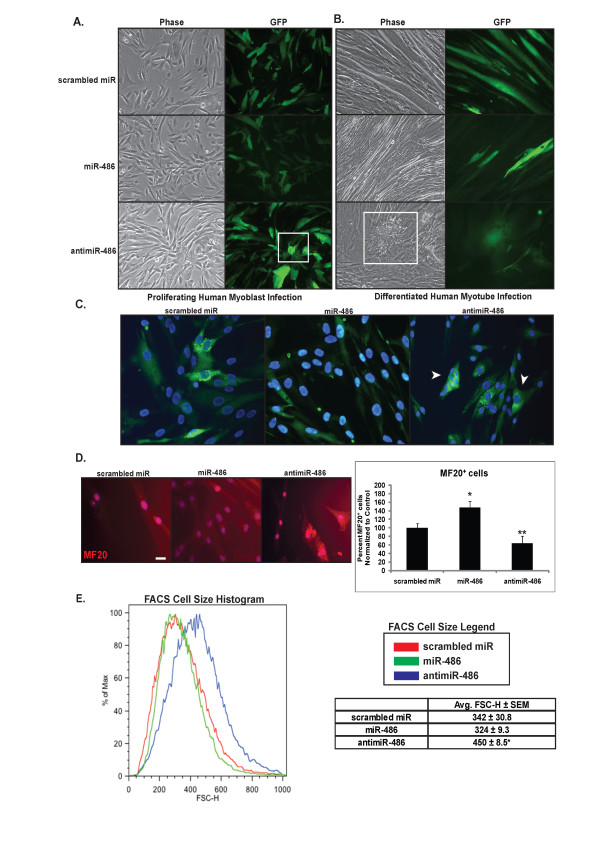
**Overexpression and inhibition of miR-486 causes profound cellular changes in myoblasts and myotubes**. **(A) **Phase contrast and GFP images of normal human myoblasts overexpressing miR-486, anti-miR-486 and a scrambled miRNA negative control virus are shown. Note that the myoblasts overexpressing the miR-486 inhibitor (anti-miR-486) appear rounded and have not flattened out (inset). **(B) **Day 4 differentiated normal human myotubes infected with the same lentiviral vectors are shown. Note the aggregate of clumped myotubes (inset) in cells infected with the anti-miR-486 lentivirus. **(C) **Higher magnification (×40) of normal human myoblasts infected with lentivirus expressing scrambled miRNA, miR-486 and anti-miR-486. The cells show detection of the GFP reporter and 4',6-diamidino-2-phenylindole (DAPI) to detect the DNA. Note that the anti-miR-486-infected myoblasts are rounded (arrowheads) and increased in overall size compared to the scrambled miRNA and miR-486-overexpressing myoblasts. **(D) **MF20 (red) staining of normal human myoblasts overexpressing scrambled miRNA, miR-486 or anti-miR-486 lentivirus. Chart indicates reduced levels of MF20^+ ^myoblasts that express anti-miR-486 lentivirus; conversely, myoblasts with high levels of miR-486 have increased amounts of MF20^+ ^myoblasts. Ten fields of GFP-positive (not shown) and DAPI-positive cells were counted for the presence of MF20^+ ^staining. Values were normalized to the scrambled miRNA. *P < 0.005 and **P < 0.05. Scale bar indicates 35 μm. **(E) **FACS histogram reveals an increase in cell size in myoblasts infected with anti-miR-486 lentivirus. The *x*-axis represents forward scatter height (FSC-H), and the *y*-axis represents the percentage cell size relative to the control scrambled miRNA myoblasts. FSC-H is quantified in the table that shows that anti-miR-486 (blue line) have increased in cell size compared to myoblasts overexpressing miR-486 (green line) and scrambled miRNA controls (red line). *P = 0.028 ± SEM.

To identify what function miR-486 might play in normal human myoblasts, we infected cells with a lentivirus that would either overexpress miR-486 precursor miRNA, express a miR-486 inhibitor (anti-miR-486) or a scrambled miRNA-negative control virus (Figure 2). All viruses coexpressed an internal ribosome entry site (IRES)-GFP tag as an indicator of positively infected cells or myotubes. Myoblasts expressing either scrambled miRNA or high levels of miR-486 showed no visible physiological or significant structural differences at both the myoblast and myotube stages of differentiation (Figures 2A and 2B). However, in normal human myoblasts in which miR-486 was inhibited, the myoblasts showed increased size and failed to flatten out as they divided and expanded across the plate. Additionally, myoblasts expressing the anti-miR-486 inhibitor had reduced amounts of MF20^+ ^cells (myosin heavy chain) as they began to elongate and fuse to form myotubes (Figure 2D). Conversely, myoblasts that had increased levels of miR-486 had significantly more MF20^+ ^cells than the scrambled miRNA control infected myoblasts (Figure 2D). Most myoblasts in which miR-486 was inhibited showed little elongation and had increased cellular size compared to those overexpressing miR-486 and control infected myoblasts as quantified by fluorescence-activated cell sorting (FACS) analysis (Figure 2E). By day 4 postinfection, the myotubes infected with the miR-486 inhibitor virus began to break down and form large cellular aggregates (inset in Figure 2B). Therefore, in cell cultures, it appears that a reduction in miR-486 levels results in myotube destruction analogously to what may happen in the skeletal muscles of DMD patients.

### miR-486 is essential for normal myoblast fusion, cellular kinetics and viability

**Figure 3:**
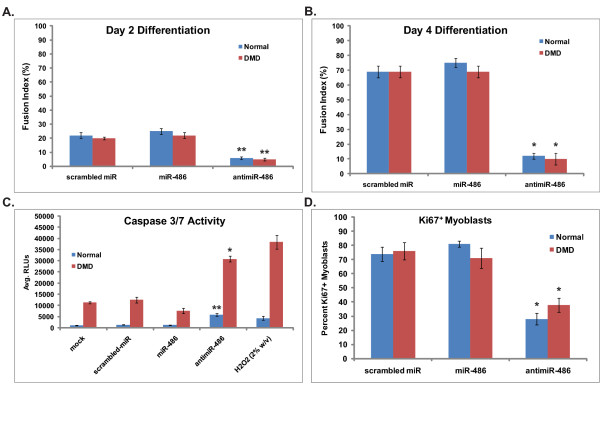
**miR-486 is essential for normal myoblast fusion, cell cycle kinetics and viability in human skeletal myoblasts**. **(A) **and **(B) **Myoblast fusion assay reveals significantly lower fusion (shown as Fusion Index % of cells with 2 or more nuclei) in miR-486-inhibited (anti-miR-486) cells. Normal and DMD myoblasts were infected at 50% confluency with lentivirus expressing miR-486, miR-486 inhibitor (anti-miR) or a scrambled miRNA control virus and allowed to differentiate to 90% confluency. Note the decreased levels of fusion in both the normal and DMD myoblasts at two time points (day 2 (Figure 2A) and day 4 (Figure 2B) of differentiation) when infected with anti-miR-486. **(C) **Increased cellular apoptosis measured using a caspase 3/7 enzymatic assay in normal and DMD myoblasts overexpressing (miR-486) or inhibiting (anti-miR-486) miR-486. DMD myoblasts overall had significantly higher caspase 3/7 activity compared with normal human myoblasts across all samples. The *y*-axis indicates average RLUs (that is, caspase 3/7 activity), and the *x*-axis indicates the condition of the myoblasts. Mock and scrambled miRNA-GFP-infected myoblasts served as negative controls (*n *= 3 replicates/cohort). Cells treated with hydrogen peroxide (H_2_O_2_) for 12 hours served as positive controls. **(D) **Ki-67 levels measured by immunofluorescence in normal and DMD myoblasts which have increased miR-486 expression, reduced miR-486 expression or expression of scrambled miRNA (negative control). Note the decreased levels of Ki-67 in myoblasts in which miR-486 expression is knocked down. *P < 0.005 and **P < 0.05 for comparisons between scrambled miRNA (control) versus miR-486- and/or anti-miR-486-infected myoblasts.

To identify what effect manipulating miR-486 expression levels in myoblasts and myotubes might have on cellular kinetics, fusion and viability, miR-486 expression was manipulated in both normal and DMD myoblast cell lines. Both cell types were then assayed for various cellular markers of myoblast fusion index, cellular viability and cell cycle kinetics. Normal human myoblasts showed increased proliferation and a higher fusion index while expressing higher levels of miR-486 at earlier time points compared with scrambled miRNA controls. By day 2 postdifferentiation, the normal human myoblasts expressing miR-486 had amounts of multinucleated myotubes similar to those of the control scrambled miRNA myoblasts, whereas the anti-miR-486-expressing myoblasts had significantly reduced amounts of multinucleated myotubes (Figures 3A and 3B). When challenged with a scratch wound assay, myoblasts infected with lentivirus that caused overexpression of miR-486 migrated at a faster rate and closed the wound almost completely after 12 hours compared to control myoblasts expressing scrambled miRNA (Additional file 2, Figure S2). Conversely, myoblasts infected with a lentivirus anti-miR-486 inhibitor failed to migrate following the scratch wound compared to scrambled miRNA controls (Additional file 2, Figure S2). These results suggest that miR-486 plays a role in the migration of myoblast progenitor cells and that alteration of miR-486 expression affects the signaling pathways essential for cellular migration. To determine the role that miR-486 might play in cellular viability, caspase-3/7 levels were measured in myoblasts that either overexpressed or knockdown miR-486 levels using a luminescent reporter assay. Normal myoblasts expressing high levels miR-486 showed no significant differences compared to scrambled miRNA controls (Figure 3C). However, inhibition of miR-486 in normal myoblasts increased levels of caspase-3/7 and resulted in higher levels of apoptosis. In DMD myoblasts, overexpression of miR-486 again had little effect on cellular viability, whereas inhibition of miR-486 resulted in increased caspase-3/7 levels and thus increased cellular apoptosis (Figure 3C) (*P *< 0.05 for normal myoblasts and *P *< 0.005 for DMD myoblasts). This correlates with previous studies of DMD myoblasts in which researchers reported increased levels of apoptosis in dystrophin-deficient muscles marked by high caspase-3 levels [25]. Finally, we assayed for the rate of proliferation of myoblasts by quantifying the percentage of Ki-67^+ ^cells per 500 total positively infected cells. Myoblasts expressing high miR-486 or scrambled miRNA showed no differences in the percentage of Ki-67^+ ^cells; however, both normal and DMD anti-miR-486-expressing myoblasts showed reduced amounts of Ki-67^+ ^cells (Figure 3D). These results imply that inhibition of miR-486 has detrimental effects on the ability of myoblasts to proliferate and maintain normal cellular kinetics. miR-486 expression in myoblasts most likely is expressed at a threshold level necessary for myoblast cellular viability, which is compromised in the absence of a functional dystrophin protein.

### miR-486 regulates a variety of PTEN/AKT signaling components in skeletal muscle

**Figure 4:**
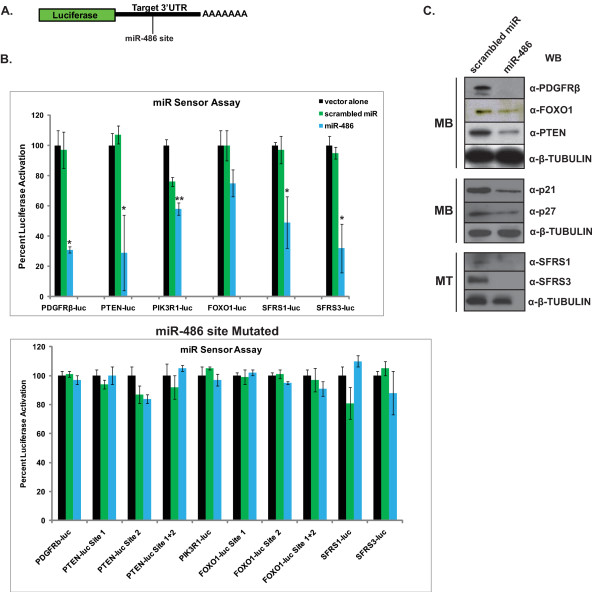
**Members of the PTEN/AKT signaling pathway and splicing factors are direct downstream targets of miR-486 in skeletal muscle**. **(A) **Schematic shows the location of the 3'UTR miR-486 target cloned downstream of the luciferase gene reporter with a poly(A) tail. **(B) **Luciferase miRNA reporter assay of several of the 3'UTR of the predicted miR-486 direct downstream targets. The lower graph shows results from the mutation of the miR-486 seed site, which ablates miR-486 binding and functions to derepress luciferase expression. The *y*-axis displays relative RLU activity normalized to 100% activation of the reporter transfected alone (vector alone). Error bars indicate SEM (*n *= 3 replicates, with the experiment performed on three separate occasions using HEK293T cells). *P < 0.005 and **P < 0.05. **(C) **Western blots of myoblasts overexpressing miR-486 or scrambled miRNA controls. Anti-β-tubulin served as a loading control. Western blot analysis of alternately splicing factors SFRS1 and SFRS3 was performed from myotube lysates from which both proteins are most abundantly expressed.

To further examine the functional role of miR-486 in normal and dystrophic muscles, we utilized several bioinformatics software programs (TargetScan, http://www.targetscan.org/; PicTar, http://pictar.mdc-berlin.de/; and MicroCosm, http://www.ebi.ac.uk/enright-srv/microcosm/htdocs/targets/v5/) to identify those genes whose expression might be altered by miR-486 expression. Among the list of potential genes possibly modulated by miR-486 expression, only those that were evolutionarily conserved across human and mouse genomes were selected. An additional preference was given to genes with known expression in skeletal muscle and/or stem cells, as well as to genes previously demonstrated to be significantly dysregulated in our previous dystrophin-deficient skeletal muscle microarray analyses of RNA expression [10,26]. Several genes that were possible targets for miR-486 were identified, including *PTEN*, *PDGFRβ*, *FOXO1*, *IGF-1*, *PIK3R1 *(p85α), *SFSR1 *and *SFSR3 *(Additional file 3, Figure S3). The 3'UTR for each of the predicted transcripts that contained these miR-486 binding sites were cloned into a luciferase (luc) miRNA reporter (Figure 4A). These 3'UTR-luc constructs were transfected into human embryonic kidney HEK293T cells along with constructs that overexpress miR-486, scrambled miRNA (negative control) plasmids or the vector alone. Overexpression of miR-486 resulted in significant decreases in the luciferase activity in the PDGFRβ-luc, PTEN-luc, PIK3R1-luc, SFSR1-luc and SFSR3-luc reporter plasmids (Figure 4B). FOXO1-luc levels were slightly decreased, but not at statically significant levels (Figure 4B). Further validation of these seed sites as binding sites for miR-486 was done by mutating several conserved bases of the eight-nucleotide seed site in the 3'UTR-luc reporters, which resulted in restoration of luciferase levels to those of scrambled miRNA and untransfected controls (Figure 4B). miRNA repression of genes can occur by either mRNA degradation or inhibition of mRNA translation, and it is necessary to measure the protein levels of genes containing the miR-486 seed sites. To measure the protein levels, normal human myoblasts were infected with lentiviruses expressing miR-486 along with an IRES-GFP reporter to indicate infection efficiency. In addition, all miR-486 targets were verified at the protein level by Western blot analysis. Overexpression of miR-486 resulted in significantly decreased protein levels of PTEN, PDGFRβ, FOXO1, SFSR1 and SFSR3 (Figure 4C). Several of these proteins comprise the PTEN/AKT pathway, which is essential for normal cellular proliferation through the regulation of the critical cyclin-dependent kinase inhibitors CDKN1A (p21) and CDKN1B (p27) through FOXO1-dependent transcriptional regulation [27,28]. Overexpression of miR-486 in myoblasts also decreased the levels of the FOXO1 targets p21 and p27 (Figure 4C). Given that miRNA are known to regulate several components in the same pathway, it seems likely that miR-486 acts on the p21/FOXO1 pathway through its primary regulation of the PTEN/AKT pathway.

### Transgenic mice expressing miR-486 have impaired skeletal muscle regeneration following CTX injury

**Figure 5:**
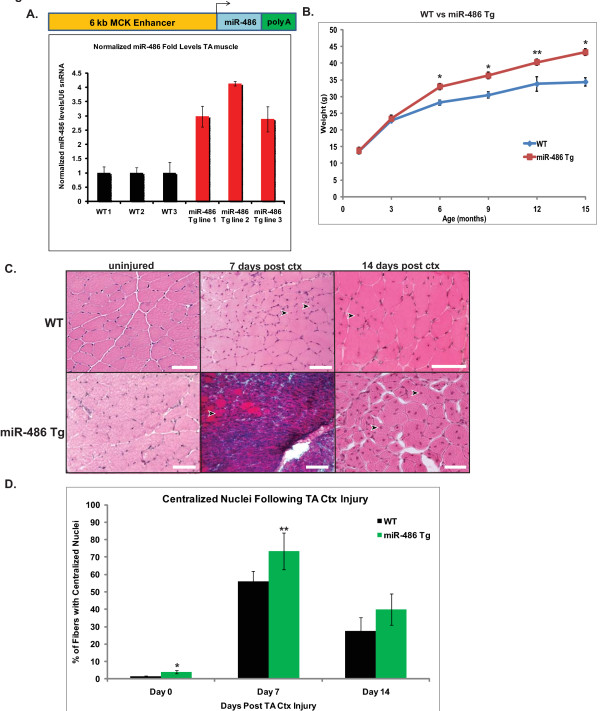
**Transgenic miR-486 overexpression in mice results in abnormal skeletal muscle regeneration following CTX-induced TA injury**. **(A) **Diagram indicating the construct used to generate MCK-miR-486-transgenic mice. Real-time qPCR of miR-486 expression levels in the TA muscle was analyzed from three separate wild-type (black bars) and miR-486 Tg (red bars) adult mice. Expression levels were normalized to U6 snRNA levels and compared to wild-type (control) levels. **(B) **Mouse weights of wild-type and miR-486 Tg mice versus age (in months) are shown. Three mouse cohorts of male and female littermates were weighed from 1, 3, 6, 9, 12 and 15 months of age (*n *= 5 male or female mice per cohort). *P < 0.005 and **P < 0.05. **(C) **H & E-stained histological sections of wild-type and miR-486 Tg mice on day 0 (uninjured), 7 and 14 post-CTX-induced TA injury. Arrowheads indicate centralized myonuclei. Scale bars = 20 μm. **(D) **Percentages of total centralized myonuclei during CTX-induced TA injury in wild-type and miR-486 Tg mice. Percentages are based on counts of 200 myofibers in three separate mice per cohort at three time points (0, 7 and 14 days post-CTX injury). *P < 0.005 and **P < 0.05.

To characterize miR-486 function *in vivo*, transgenic mice that overexpress miR-486 transcript at levels greater than twofold compared to their wild-type control littermates were generated (Figure 5A). To develop these mice, a DNA construct containing the mouse miR-486 genomic sequence was cloned downstream of the muscle CK (MCK) enhancer element, which is expressed exclusively in the heart and skeletal muscle myofibers, thus excluding muscle satellite cells [29]. The MCK miR-486-transgenic (miR-486-Tg) mice were viable and displayed no overt phenotypic differences from their wild-type littermate controls at six months of age, other than a slight increase in weight that was maintained for the remainder of their lifespan (Figure 5B). To test whether increased expression of miR-486 would influence *in vivo *regeneration of muscle, similar to our observations in cell culture, the miR-486-Tg mice were injected with CTX and their regenerative capacity was compared to the injured muscles of their wild-type littermates. At seven days postinjury, compared to wild-type day 7 CTX controls, the muscles of the miR-486-Tg mice had failed to fully regenerate and had accumulations of myonuclei (Figure 5C). By day 14 post-CTX injury, the miR-486-Tg mice had recovered their muscle fiber architecture, but many centralized, multinucleated myofibers remained, whereas significantly fewer centralized myonuclei were observed in the day 14 wild-type controls (Figure 5D). Immunofluorescent (IF) staining of the proliferation marker Ki-67 revealed that the miR-486-Tg mice had significantly higher amounts of Ki-67^+ ^cells at days 7 and 14 post-CTX injury compared to their wild-type controls (Additional file 4, Figure S4). Together these results implicate a delay in skeletal muscle regenerative capacity in mice that maintain increased miR-486 levels and further implicate miR-486 as a potential indirect regulator of muscle satellite cell kinetics and fusion capabilities during muscle regeneration.

### Transgenic mice overexpressing miR-486 have altered levels of PTEN/AKT signaling components during skeletal muscle regeneration

**Figure 6:**
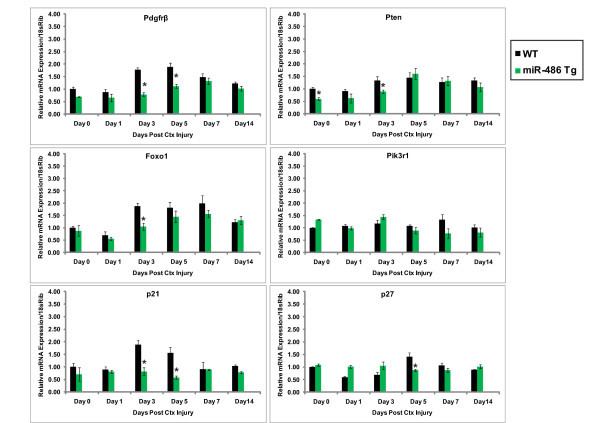
**miR-486 overexpression regulates skeletal muscle PTEN/AKT signaling *in vivo***. Real-time qPCR measuring levels of Pdgfrβ, Pten, Foxo1, Pik3r1 (p85α), p21 (Cdkn1a) and p27 (Cdkn1b) in mouse skeletal muscle during the time course of skeletal muscle regeneration (day 0 (uninjured) to day 14 post-CTX TA injection) in wild-type (black bars) and miR-486 Tg (green bars) mice. Three muscles per time point per day of injury were analyzed in triplicate. Error bars indicate SEM. Relative mRNA expression levels were normalized to 18 s ribosome (18sRib) loading control and day 0 (uninjured) wild-type muscle. *P < 0.05 wild-type vs. miR-486 Tg values.

To fully evaluate the functional role of miR-486 *in vivo *by real-time qPCR, the CTX-injured TA muscles of miR-486-transgenic and wild-type mice were used to measure the expression levels of genes known to be critical components of the PTEN/AKT pathway. The miR-486-transgenic mice showed significantly decreased mRNA expression of the miR-486 downstream target genes (mRNA for *Pdgfrβ *and *Foxo1*) in their skeletal muscles at specific time points during days 3 through 7 post-CTX injury as would be expected if these genes were direct miR-486 targets (Figure 6). *Pik3r1 *showed decreased mRNA expression on days 5 through 14 post-CTX-induced injury, but not at significant levels. *Pten *levels remained reduced in the uninjured (day 0) miR-486-Tg muscles and at day 3 of muscle regeneration (Figure 6). The Forkhead transcription factor Foxo1 is the downstream target of PTEN/AKT signaling and regulates the cell cycle through transcriptional activation of several cyclin-dependent kinase inhibitors (such as p21 and p27) in many cell types, including skeletal muscle [28,30,31] (Figure 6). At the same critical time point during skeletal muscle injury (days 3 through 5) in the miR-486-Tg mice, there was a reduction in the levels of the Foxo1 targets p21 and p27, but not p57 (Figure 6 and Additional file 5, Figure S5), which is not a direct target of Foxo1 signaling [32] but has been shown to play an essential role in skeletal muscle formation [33]. Additionally, several Foxo1-interacting factors regulating cellular viability, such as *Bcl2*, *Bax *and *Bim*, showed slight modulations in gene expression during the same critical time points (days 3 through 7 post-CTX injury) during skeletal muscle regeneration (Additional file 5, Figure S5). Together these studies implicate miR-486 as a negative regulator of the PTEN/AKT signaling components and their downstream effector proteins *in vivo *during skeletal muscle regeneration.

## Conclusions

**Figure 7:**
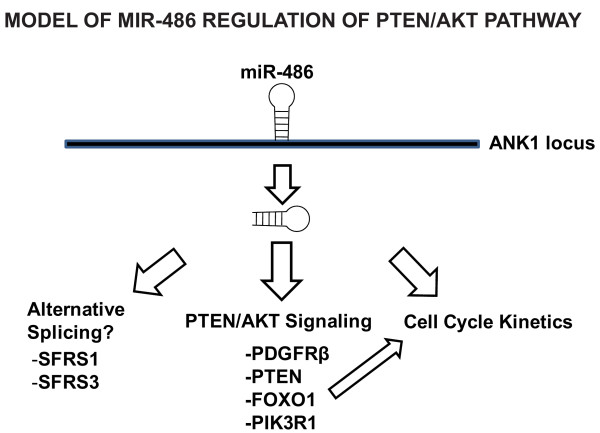
**miR-486 is an important regulator of PTEN/AKT signaling in normal and dystrophic muscle**. Schematic of miR-486 regulation of PTEN/AKT pathway upstream and downstream signaling components in skeletal muscle is shown. Regulation of the PTEN/AKT pathway in turn regulates FOXO1, which is a well-characterized regulator of p21 and p27 in skeletal muscle.

In summary, these studies identify miR-486 as a uniquely downregulated miRNA in DMD. With many targets of miR-486 residing in the PTEN/AKT signaling pathway (both upstream and downstream), it is likely that miR-486 functions as a regulator of cell cycle kinetics, cell cycle viability and perhaps cellular migration in skeletal muscle (Figure 7). Additionally, our identification of miR-486 as a potent repressor of several components of the PTEN/AKT signaling pathway follows, as many of these signaling components are shown to be upregulated in DMD muscle while miR-486 is expressed at reduced levels. Another recent study profiling dysregulated miRNA in *mdx *mouse muscle demonstrated that the lack of dystrophin resulted in the dysregulation of several miRNA, including miR-486, which further validates our findings that miR-486 is reduced in the absence of a functional dystrophin. Although the *mdx^5cv ^*mutant mouse has a less severe phenotype than that observed in human DMD patients, it contains frame shift mutations resulting from an A to T transversion in exon 10 of the dystrophin gene that results in a 53-bp deletion sequence starting from exon 10 and does not produce the large Dp427 skeletal muscle isoform [34]. Thus, one might hypothesize that the presence of the large Dp427 skeletal muscle isoform is essential for maintaining normal miR-486 expression levels, especially since BMD patients with a partially functional truncated dystrophin protein [3] have normal miR-486 regulation in muscle, which we have validated in this study.

miR-486 has also been shown to be dysregulated in three other pathological conditions: white blood cells during sepsis, glioblastomas and lung adenocarcinomas [35-37]. Many cancers have been shown to have dysregulated PTEN/AKT signaling resulting from a failure to regulate normal cell cycle kinetics and cellular apoptosis [38,39]. miR-486's influence on cell cycle progression through the regulation of PTEN/AKT signaling components and their subsequent downstream regulation of cyclin-dependent kinase inhibitors reinforces the role of miR-486 as a biomarker in several cancers. In cardiomyocytes, miR-486 has been shown to be a negative regulator of PTEN/AKT signaling during heart ventricle postnatal development [20]. That study also demonstrated that miR-486 expression can be activated by myocardin-related transcription factor A (MRTF-A, also referred to as MAL/MKL1) [20], a coregulator of the serum response transcription factor, which has been shown to regulate actin polymerization and Rho signaling in cardiac hypertrophy [40,41]. Although MRTF-A has been studied predominately for its role in cardiac hypertrophy and heart failure, it is expressed in C_2_C_12 _myoblasts and may be essential for normal myogenic differentiation. Additionally, MRTF-A may have a function in skeletal muscle hypertrophy and atrophy through a striated activator of Rho signaling-dependent pathway [42,43]. Another recent study identified miR-486 along with miR-206 (another muscle-enriched miRNA) as a regulator of myoblast cell cycle kinetics through its downregulation of Pax7 mRNA and another muscle-enriched miRNA, miR-206 [44]. However, given that miR-486 expression is activated by myogenic factors that are downstream of Pax7 (such as MyoD1), it is likely that miR-486's regulation of PTEN/AKT signaling might explain the cell cycle defects due to the Pax7^+ ^low expression in MyoD1^+ ^myoblasts [45,46].

miR-486 may also have important regulatory functions that are independent of PTEN/AKT signaling in other cell types and pathological diseases. Recently, miR-486 has been found to be significantly upregulated in lymphoblast cell lines derived from autistic patients [47]. Intriguingly, patients with DMD have an increased incidence of autism or other neurological disorders [48,49]. Given that two of the components that are essential for normal alternative splicing of mRNA (splicing factor, arginine/serine-rich 1 (SFRS1) and SFRS3) are likely targets of miR-486, it is possible that miR-486 could play a role in the translation of genes essential for normal cellular homeostasis in other tissues, such as the brain.

These studies offer a possible mechanism for the upregulation of PTEN/AKT signaling pathway components, which have previously been observed in dystrophin-deficient skeletal muscle. Recently, it has been shown that mice lacking PTEN had improved skeletal muscle regeneration when fed a high-fat diet [50]. Modulation of the PTEN/AKT signaling pathway through miR-486 expression has the potential to be a novel therapy for treating DMD. Delivery of miR-486 in skeletal muscle might have a beneficial effect in ameliorating the secondary signaling defects that are observed in dystrophin-deficient muscle. Advances in the use of stable *in vivo *exon-skipping morpholinos for the correction of out-of-frame *DMD *gene mutations and deletions have led to an increased interest in the use of nonintegrative methods for the treatment of DMD [51,52]. One can envision that a combination therapy involving the overexpression of intramuscular injection of stabilized miR-486 along with exon-skipping morpholinos might restore muscle function and prevent some of the muscle loss observed in DMD patients.

## Materials and methods

### Human biopsies and establishment of primary myoblast cell lines

DMD/BMD patient muscle tissue was obtained during either diagnostic skeletal muscle biopsies or other clinical surgeries. Control muscle tissue was obtained from patients undergoing clinical orthopedic surgery. Each biopsy sample was divided into two either to be frozen for sectioning or to be used to establish myoblast cultures. All patients were males between 6 and 65 years of age and were classified as normal, DMD or BMD based on a combination of DNA sequencing, dystrophin protein analysis by Western blotting and physician evaluation. The muscle groups biopsied were from human TA, back (latissimus dorsi) or hip muscles (vastus lateralis), depending on tissue availability. Mutations in the *DMD *gene locus were validated by the clinical DNA Diagnostic Center at Children's Hospital Boston by both Southern blot analysis and automated sequencing analysis using universal conditions for direct sequencing methods [53]. All BMD samples contained separate deletions residing in exons 45 through 49 of the dystrophin gene (patient 1, exons 45 through 48 deleted; patient 2, exons 48 and 49 deleted; and patient 3, exons 45 through 49 deleted), which resulted in a truncated but partially functional dystrophin protein. All BMD muscle biopsies were analyzed by Western blotting and were determined to have truncated dystrophin protein (Dp427) molecular weights that ranged from 380 and 402 kDa. All patients gave their written and oral consent prior to surgery, and all protocols were approved by the Children's Hospital Boston human subjects internal review board (protocol 03-12-205R).

Myoblasts were isolated from fresh biopsies by first washing the samples in ice-cold 1× PBS and then mincing them with sterile scalpels on a 100-mm plate for 10 minutes until all clumps were removed. The minced muscles were then incubated with a collagenase IV neutral protease (Worthington Biochemical Corp. Lakewood, NJ, USA) at a ratio of 3.5× the gram weight of the minced muscle. The plate was incubated at 37°C for 45 minutes until the cells were in suspension by pipetting the mixture thrice at 15-minute intervals. Ten milliliters of human skeletal muscle growth medium (PromoCell, Heidelberg, Germany) were added to the mixture. The mixture of cells was then passed through a 70-μm filter and centrifuged at 514 × *g *at 4°C for 10 minutes. The supernatant was aspirated, and then the cells were resuspended in red blood cell lysis buffer (Qiagen, Valencia, CA, USA) and incubated for three minutes at room temperature. Following incubation, 22 mL of growth medium were added to the cells, followed by a second filtration using a 40-μm filter. The filtrate was then centrifuged at 514 × *g *at 4°C for 10 minutes. The supernatant was aspirated, and then the cells were resuspended in 10 mL of growth medium before being seeded onto uncoated 100-mm plates for one hour in a 37°C incubator. Following the one-hour preplating on uncoated plates, the supernatants containing unattached cells enriched for myoblasts were replated onto 0.1% gelatin-coated 100-mm plates (porcine; Sigma-Aldrich, St Louis, MO, USA). Cells were passaged every two to three days. Additionally, the cells were subjected to at least two additional preplating steps in which the fibroblasts adhered to the uncoated dishes and the slowly adhering myoblast fraction was then plated onto the gelatin-coated dishes, similarly to a protocol described previously [54]. Myoblast enrichment was measured by immunofluorescent labeling of cells using a desmin antibody (1:500 dilution; Epitomics Inc., Burlingame CA, USA). Cellular preparations that contained over 90% desmin-positive myoblasts were considered "myoblast enriched" and thus were appropriate for cell culture experiments.

### Lentivirus production

Lentivirus vectors that stably overexpress a pre-miR-486 precursor, anti-miR-486 (miRZipsSystem Biosciences Inc., Mountain View, CA, USA) or scrambled miRNA (nonsense small hairpin RNA) controls that contained bicistronic IRES-GFP tags were obtained commercially (System Biosciences Inc., Mountain View, CA, USA). Third-generation lentiviral packaging plasmids (MDL/RRE, Rev and VSV-G) were a generous gift from GQ Daley (Children's Hospital Boston) [55]. HEK293T cells were grown in 100-mm plates in 10% fetal bovine serum (FBS)/DMEM (Mediatech Inc., Manassas, VA, USA) supplemented with 1× antibiotics (Invitrogen, Carlsbad, CA, USA). Lentiviral vectors along with packaging plasmids were transfected in ten 10-cm plates (at a ratio of 3 μg lentiviral vector to 1 μg of each packaging plasmid per 10-cm plate) containing 90% confluent HEK293T cells using Lipofectamine 2000 reagent (Invitrogen). Three days after transfection viral supernatants were pooled and filtered through 45-μm filters (VWR International LLC., Radnor, PA, USA), mixed with a 1/3^rd ^volume of Lenti-X lentivirus concentrator (Clontech Laboratories Inc., Mountain View, CA, USA) and incubated overnight at 4°C. The following day the lentiviral concentrate elute was pelleted by centrifugation at 1,500 × *g *for 45 minutes at 4°C. Viral pellets were resuspended in DMEM at one-twentieth the starting volume. The lentiviruses were titered by calculating the percentage of GFP fluorescence to the total number of plated cells in subsequent tenfold serial dilutions following infection of 500,000 HEK293T cells [56]. Most lentiviral infections were done at a viral titer of approximately 2 × 10^8 ^TU/mL.

### Mouse lines

C57BL6/J wild-type control mice and *mdx^5cv ^*mice were purchased from Jackson Laboratories (Bar Harbor, ME, USA) [57]. All mouse husbandry and transgenic lines were approved by the Children's Hospital Boston Animal Facilities/Institutional Animal Care and Use Committee and maintained in pathogen-free cages following federal standard of care practices. Individual mice were weighed on a Denver Instrument DLT Series scale (Denver Instrument, Bohemia, NY, USA All mice were fed a 5P00 Prolab RMH 3000 standard chow diet (5% fat; LabDiet, PMI Nutrition International, St. Paul, MN, USA;) and had access to food *ad libitum*. All mouse lines and subsequent breeding were maintained on a C57BL6/J background under the Children's Hospital Boston ARCH animal protocol (10-12-1852R).

The MCK-miR-486-Tg overexpression mouse lines were generated using the pBS-MCK backbone plasmid obtained from Addgene (plasmid 12528; Addgene, Cambridge, MA, USA). The MCK plasmid contains a 6-kb enhancer region for the mouse muscle creatine kinase *Ckm *gene and has been previously characterized [29,58]. A 500-bp fragment containing the stem loop precursor sequence of mouse miR-486 was amplified by PCR from C57BL6/J mouse genomic DNA and subcloned into the *Pst*I/*Spe*I restriction sites of the pBS-MCK plasmid. The 6-kb MCK fragment plus the miR-486 sequence and a simian virus 40 (SV40) polyadenylation (poly(A)) signal were excised from the plasmid using *Kpn*I/*Sac*I restriction enzymes. Following DNA purification using the GeneClean II kit (MP Biomedicals LLC, Solon, OH, USA), the linearized DNA fragment was then injected into the pronucleus of C57BL6/J fertilized eggs and transplanted into the uterine horn of pseudopregnant ICR foster mice. A total of 55 viable pups from five separate injections were genotyped by PCR using primers specific for the MCK enhancer element and the SV40 poly(A) signal transduction sequence. All F_0 _founder mice were maintained on a C57BL6/J background. Additional mouse genotyping was performed commercially by Transnetyx, Inc. (Cordova, TN, USA).

### Additional cell cultures

HEK293T cells were a generous gift from the laboratory of GQ Daley (Children's Hospital Boston). The cells were maintained using 10% FBS (Atlanta Biologicals Inc., Lawrenceville, GA, USA)/DMEM (Invitrogen) supplemented with antibiotics (Antibiotic-Antimycotic; Invitrogen) as described previously [55].

### miRNA isolation and real-time qPCR

miRNA and mRNA were extracted from either cell lines or animal tissue using the miRVana Isolation kit (Ambion Austin, TX, USA). Fifty nanograms of total enriched miRNA per sample were copied into cDNA (through RT) using the Multiscribe RT System (Applied Biosystems Carlsbad, CA, USA**)**. One microliter of RT reaction was used for each specific miRNA TaqMan assay (Applied Biosystems), which was amplified using No-Amp Erase TaqMan Polymerase (Applied Biosystems) according to the manufacturer's instructions. All samples were analyzed in 96-well optical plates on an ABI 7900HT real-time PCR machine (Applied Biosystems). Cycle times were normalized to U6 small nuclear RNA as a loading control. Relative fold expression and changes were calculated using the 2^-ΔΔCt ^method [59].

Total RNA from muscle biopsies and cell cultures was extracted using the mirVana miRNA/mRNA Isolation Kit (Ambion). One microgram of total RNA was copied into cDNA using the First-Strand cDNA Synthesis Kit (Invitrogen). Tenfold serial dilutions of cDNA were analyzed on 96-well optical plates using Power SYBR Green Master Mix (Applied Biosystems) and analyzed on the ABI 7900HT real-time PCR machine. Primers specific for each gene were designed using National Center for Biotechnology Information Primer-Blast http://www.ncbi.nlm.nih.gov/tools/primer-blast/index.cgi?LINK_LOC=BlastHome, and all cycle time values were normalized to an 18 s ribosome loading control. All real-time qPCR primers are listed in Additional file 6, Table S1.

### Antisera

The following primary antibodies were used in this study: anti-GFP (1:1,000 dilution Western blot; MBL International Corp. Woburn, MA, USA; and 1:50 dilution IF; Chemicon International Temecula, CA, USA), anti-β-tubulin horseradish peroxidase (HRP) (1:1,000 dilution Western blot; Cell Signaling Technology Danvers, MA, USA), anti-PTEN (1:1,000 dilution Western blot; Cell Signaling Technology), anti-PDGFRβ (1:1,000 dilution Western blot; Bioworld Technology Inc. St. Louis Park, MN, USA), anti-PIK3R1 (1:1,000 dilution Western blot; Abcam), anti-FOXO1 (C29H4 clone, 1:1,000 dilution Western blot; Cell Signaling Technology), anti-SFRS1 (1:1,000 dilution Western blot; Aviva Systems Biology San Diego, CA, USA), anti-SFRS3 (clone 2D2, 1:1,000 dilution Western blot; Sigma-Aldrich), anti-MF20 (1:25 dilution IF; Developmental Studies Hybridoma Bank, Iowa City, IA, USA); anti-Ki-67 (1:200 dilution IF; BD Pharmingen Inc., San Diego, CA, USA), anti-desmin (1:1,000 dilution IF Epitomics Inc. Burlingame CA, USA;), anti-p21 (1:1,000 dilution Western blot; Abcam) and anti-p27 (1:500 dilution Western blot; Abcam). Secondary antibodies used for Western blot analysis included anti-mouse and anti-rabbit HRP-conjugated antibodies (1:2,000 dilution Western blot; Cell Signaling Technology), along with anti-goat HRP-conjugated antibody (1:1,000 dilution Western blot; Santa Cruz Biotechnology, Santa Cruz, CA, USA). Secondary antibodies (Invitrogen) were used for immunofluorescent detection of primary antisera in mouse and rabbit with Alexa Fluor fluorochromes conjugated at 488 nm and 565 nm.

### FACS analysis

Myoblasts were sorted using FACS analysis for detection of the IRES-GFP reporter indicating a positively infected cell and were stained with propidium iodide (2 μg/mL; BD Biosciences San Jose, CA, USA) to exclude dead cells. The myoblasts were sorted on a FACSVantage flow cytometer (BD Biosciences), and all FACS plots were analyzed using FloJo version 6.4.7 software (Tree Star, Inc., Ashland, OR, USA).

### Western blot analysis

All protein preparations were quantified using a bicinchoninic acid protein assay kit (Pierce Biotechnology Rockford, IL, USA). Thirty micrograms of whole cell protein lysate were resolved on 4% to 20% gradient Tris-acetate gels (Invitrogen). Following transfer to polyvinylidene fluoride (PVDF) membranes (Invitrogen), the membranes were blocked with 5% nonfat milk (Cell Signaling Technology)/Tris-buffered saline (TBS)-Tween 20 (Sigma-Aldrich) before being incubated overnight at 4°C with primary antisera diluted in 5% milk/TBS-Tween 20 (Boston BioProducts Inc., Ashland, MA, USA). The membranes were subjected to a series of three TBS-Tween 20 washes. The secondary HRP-conjugated antibodies were diluted in 5% milk/TBS-Tween 20 prior to being incubated with PVDF membranes (Invitrogen) for one hour at room temperature. Following additional washes prior to application of chemiluminescent substrate (Millipore, Billerica, MA, USA), the membranes were exposed on Kodak BioMax film (Sigma-Aldrich) using varying exposure times.

### CTX injections and histology of mice

Four-month-old male C57BL6/J and miR-486-Tg littermate mice were subjected to injections into their TA muscles with 10 μM CTX (C9759; Sigma-Aldrich) that was resuspended in 1× PBS. Another cohort received 1× PBS sham (no CTX) control TA injections. All mice were monitored for signs of pain or distress following the injections. The mice were killed by CO_2 _asphyxiation, and their muscles were harvested at 0 (uninjured), 1, 3, 5, 7 and 14 days post-CTX injury. The harvested muscle biopsies were then embedded in Tissue-Tek O.C.T. compound (Sakura Finetek U.S.A. Inc., Torrance, CA, USA) and snap-frozen in liquid nitrogen chilled in isopentane (Sigma-Aldrich). The frozen muscles were then cryoembedded and sectioned on a cryostat in 7-μm-thick sections. A portion of each muscle was harvested for total RNA extraction at a later time point. The muscle sections were then placed on polylysine slides. Sections were methanol-fixed for 3 minutes at -20°C and then stained with H & E (Sigma-Aldrich) to observe morphology. Sections were imaged on a Nikon Eclipse E1000 microscope (Nikon Instruments, Melville, NY, USA using SPOT imaging software (SPOT Imaging Solutions, Sterling Heights, MI, USA**)**. Some images were later modified using Adobe PhotoShop and Adobe Illustrator CS4 (Adobe Systems Inc., San Jose, CA, USA to enhance image resolution and add scale bars.

### Myoblast wound migration assay

Primary human myoblasts were seeded onto 0.1% gelatin-coated 20-mm two-well chamber slides at 25,000 cells/well. Twenty-four hours later the cells were infected with lentivirus or a 1× PBS mock control at 10 multiplicities of infection. Twenty-four hours postinfection the cell wells were scratched vertically with a P100 pipette. The cells were at 85% to 90% confluence at the time of the scratch wound. Cell migration was measured at 0, 12 and 24 hours. By 24 hours, all but the anti-miR-486-infected cells had fully migrated to close the scratch wound (data not shown). All scratch wounds were performed in triplicate.

Each group of infected cells was fixed at each of the time points with 4% paraformaldehyde for 10 minutes at 4°C with gentile agitation. Following fixation the cells were washed twice in 1× PBS and three times in 0.1% Triton X-100/1× PBS, and then they were incubated in blocking buffer (10% horse serum (Gibco, Grand Island, NY, USA) and 0.1% Triton X-100 (Sigma-Aldrich) mixed in 1× PBS, Mediatech, Inc. Manassas, VA, USA) for one hour at room temperature with light shaking. Following blocking the cells were incubated with phalloidin conjugated to Alexa Fluor 546 (1:100 dilution in blocking buffer; Invitrogen) overnight at 4°C with gentle shaking. Cells were then washed three times with 0.1% Triton X-100/1× PBS before being mounted with VECTASHIELD mounting medium containing 4',6-diamidino-2-phenylindole (DAPI) (Vector Labs, Burlingame, CA, USA). Cells that had been stained were photographed with a Nikon II(Nikon Instruments, Melville, NY, USA;) microscope using SPOT imaging software (SPOT Imaging Solutions, Sterling Heights, MI, USA) at all three time points.

### miRNA reporter assays

Following prediction of the transcripts which were thought to be targets of miR-486, the 3'UTR regions of these genes were amplified by PCR from a human total mRNA library (Ambion) or from commercially cloned constructs (GeneCopoeia Inc., Rockville, MD, USA). The 3'UTR amplicons were then cloned into the *Spe*I and *Not*I restriction sites of a modified version of the pGL2Basic vector containing a novel multicloning site from the pCR4-TOPO vector (Invitrogen) between the luciferase open-reading frame and the SV40 poly(A) signal (gift from A Dostal, J Ho and JA Kreidberg; Children's Hospital Boston).

The miRNA 3'UTR-luc reporter assay was performed by first plating 20,000 HEK293T cells/well into 48-well plates. The following day the cells were transfected using Lipofectamine 2000 reagent with 30 ng of 3'UTR miRNA-luc reporter constructs and 100 ng of miR-486 overexpression plasmid (Origene, Rockville, MD, USA); pCMVmiR-IRES-GFP vector) or scrambled miRNA controls (Origene). Forty-eight hours after transfection the cells were lysed in radioimmunoprecipitation assay buffer (Thermo Fisher Scientific Inc., Waltham, MA, USA), and 20 μL of whole cell lysate were assayed with 50 μL of luciferase substrate using the Dual Reporter Assay (Promega, Madison, WI, USA). Luciferase levels were measured on a single-tube luminometer (Berthold Technologies USA, LLC., Oak Ridge, TN, USA). The seed sites in the 3'UTR-luc reporters were mutated from GTACAGG to GgAaAtG using the QuikChange II Site-Directed Mutagenesis Kit (Stratagene, La Jolla, CA, USA).

### CASPASE 3/7 assays

Twenty-five microliters of total protein lysates from infected normal and DMD myoblasts were incubated with 100 μL of Caspase-Glo 3/7 reagent (Promega) according to the manufacturer's protocol. Luciferase measurements were taken on a single-tube luminometer (Berthold Technologies).

### Statistical analysis

Unless otherwise stated, all statistical analyses were performed using a Student's *t*-test (two-tailed).

## Abbreviations

BMD: Becker muscular dystrophy; DAPI: 4',6-diamidino-2-phenylindole; DMD: Duchenne muscular dystrophy; DMEM: Dulbecco's modified Eagle's medium; FACS: fluorescence-activated cell sorting; FSC-H: forward scatter height; GFP: green fluorescent protein; H & E: hematoxylin and eosin; IF: immunofluorescence; IRES: internal ribosome entry site; MCK: muscle creatine kinase; miRNA: microRNA; PBS: phosphate-buffered saline; qRT-PCR: quantitative reverse transcriptase polymerase chain reaction; RLU: relative light unit; SEM: standard error of the mean; snRNA: small nuclear RNA; TA: tibialis anterior; TBS: Tris-buffered saline; Tg: transgenic; U: standard unit of enzymatic activity; UTR: untranslated region.

## Competing interests

The authors declare that they have no competing interests.

## Authors' contributions

MSA and LMK designed and performed experiments and analyzed the data. JCC, NM, JAM, GK and RTG performed experiments and analyzed the data. IE performed experiments and contributed new reagents. EAE collected patient samples, family histories and reagents. PBK contributed new reagents, isolated biopsy tissues and edited the manuscript. MSA and LMK analyzed all of the data and wrote the manuscript. All authors read and approved the final version of the manuscript.

## Supplementary Material

Additional file 1 **Supplemental Figure S1. Regeneration of wild-type and *mdx^5cv ^*murine skeletal muscle following the delivery of CTX to TA muscles**. H & E staining of wild-type and *mdx^5cv ^*adult mouse TA sections of CTX-injured muscles, with uninjured TA muscles serving as controls (*n *= 3 muscles/cohort). Scale bars = 20 μm. Note the impaired differentiation and large numbers of centralized myonuclei (black arrowheads) in the *mdx^5cv ^*mice compared to their wild-type control cohorts. Click here for file

Additional file 2 **Supplemental Figure S2. miR-486 expression is essential for normal myoblast migration and wound closure**. Normal human myoblasts overexpressing lentiviral miR-486-GFP migrate faster to close the scratch wound compared with scrambled miRNA-GFP (negative control) 12 hours post-scratch wound. Myoblasts overexpressing lentiviral anti-miR-486-GFP fail to migrate to close the wound 12 hours post-scratch infliction. Top panel shows the initial scratch wound at 0 hours in phase contrast and GFP (green) fluorescence, which serves as an indicator of viral infection efficiency. Bottom panel shows myoblast wound closure 12 hours post-scratch wound in phase contrast and stained with phalloidin (red) and DAPI (DNA; blue). Representative scale bars show the diameters of the scratch wound in micrometers. Click here for file

Additional file 3 **Supplemental Figure S3. Evolutionary conservation between human and mouse seed regions of predicted miR-486 downstream target genes**. Several components of the PTEN/AKT signaling pathway are predicted targets of miR-486 in mammals (human and mouse). Shown are nine highly evolutionarily conserved miR-486 seed sites located within seven different genes: *PDGFRβ*, *FOXO1*, *PTEN*, *IGF-1*, *PIK3R1 *(p85α), *SFRS1 *and *SFRS3*. miR-486 alignment is 3' to 5', and the miR-486 binding site is 5' to 3'. Click here for file

Additional file 4 **Supplemental Figure S4. Mice that overexpress miR-486 in their muscles have increased amounts of Ki-67^+ ^cells during skeletal muscle regeneration**. Immunofluorescent staining of skeletal muscle from wild-type (WT) and miR-486 Tg mice at day 0 (uninjured), day 7 and day 14 post-CTX-induced TA injury. The graph denotes increased levels of Ki-67^+ ^myogenic cells in the miR-486 Tg mice at days 7 and 14 post-CTX-induced TA injury. The Ki-67^+ ^cells were counted based on averages taken from ten separate fields. The sections were stained with desmin (red) and Ki-67 (green) antibodies. Arrowheads indicate Ki-67^+ ^cells in a given field, and scale bars indicate 40 μm in length. All images were obtained at ×40 magnification. *P < 0.005 and **P < 0.05 wild-type vs. miR-486 Tg. Click here for file

Additional file 5 **Supplemental Figure S5. miR-486 overexpression in mouse skeletal muscle results in dysregulation of PTEN/AKT downstream signaling components**. Real-time qPCR of PTEN/AKT signaling targets (p57, Bcl2, Bim and Bax) in adult wild-type and miR-486 Tg mice during a CTX-induced skeletal muscle injury time course (days 0 to 14). Relative mRNA fold expression was normalized to 18sRib rRNA expression levels. *P < 0.05 wild-type (black bars) vs. miR-486 Tg (green bars). Click here for file

Additional file 6 **Supplemental Table S1. Real-time qRT-PCR primers used in this study**. Mouse species-specific primers that spanned an intron were used when appropriate. Click here for file
