# Supplementary figures and images for: Regulation of DMD pathology by an ankyrin-encoded miRNA
Source: Skelet Muscle. 2011 Aug 8;1:27. doi: 10.1186/2044-5040-1-27 (PMC3188430; doi:10.1186/2044-5040-1-27)

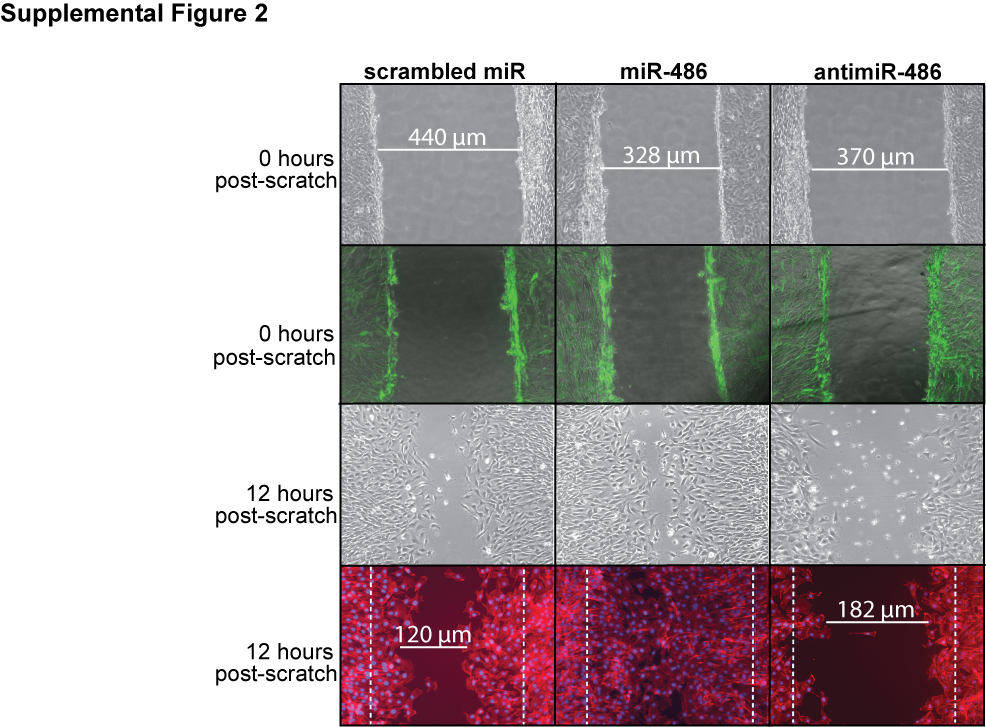

Supplement: Additional file 2 — Supplemental Figure S2. miR-486 expression is essential for normal myoblast migration and wound closure. Normal human myoblasts overexpressing lentiviral miR-486-GFP migrate faster to close the scratch wound compared with scrambled miRNA-GFP (negative control) 12 hours post-scratch wound. Myoblasts overexpressing lentiviral anti-miR-486-GFP fail to migrate to close the wound 12 hours post-scratch infliction. Top panel shows the initial scratch wound at 0 hours in phase contrast and GFP (green) fluorescence, which serves as an indicator of viral infection efficiency. Bottom panel shows myoblast wound closure 12 hours post-scratch wound in phase contrast and stained with phalloidin (red) and DAPI (DNA; blue). Representative scale bars show the diameters of the scratch wound in micrometers. [file 2044-5040-1-27-S2.PNG]
